# Blood pressure measurement technique in clinical practice in the NHS Greater Glasgow and Clyde

**DOI:** 10.1038/s41371-024-00984-5

**Published:** 2024-12-05

**Authors:** Dellaneira Setjiadi, Colin Geddes, Christian Delles

**Affiliations:** School of Cardiovascular and Metabolic Health, University of Glasgow, Glasgow, UK

**Keywords:** Diagnosis, Renovascular hypertension

## Abstract

Blood pressure (BP) measurement is a common procedure conducted in various disciplines and is widely available on clinical reports. The diagnosis and management of hypertension require reliable measurement of BP in outpatient clinics. Published studies suggest the standardised method for BP measurement is difficult to apply in routine clinical practice. This study aimed to assess the current practice of BP measurement in outpatient clinics in relevant secondary care clinical specialties across the 15 separate hospital sites of the NHS Greater Glasgow and Clyde region (population 1.2 million) compared to the recommended standardised method. An online questionnaire was developed and disseminated to the supervising clinician of each of 268 regular outpatient clinics. The questionnaire focused on the standardised BP method (patient preparation, environment, and BP measurement technique). The questionnaire was returned for 110 clinics. 73 (66.4%) of the participating clinics measure BP routinely and these formed the basis for further analysis. 3 clinics (4.1%) apply all components of the standardised BP method. 5 (6.9%) clinics deliver advice to patients prior to clinic attendance on how to prepare for BP measurement. 61 (83.6%) of participating clinics have a dedicated quiet environment for BP measurement. 50 (68.5%) clinics always place the cuff on bare upper arm and 63.0% use a cuff size appropriate to upper arm circumference. In a wide range of secondary care out-patient clinic settings, we found that BP measurement rarely adheres to the recommended standards. This has important implications for the quality of treatment decisions that are based on BP measurement.

## Introduction

Assessment of blood pressure (BP) is one of the most common measurements conducted in outpatient clinical settings, particularly in cardiology, diabetes and endocrinology, nephrology and specialist hypertension clinics. Accurate BP measurements are crucial for the diagnosis and management of hypertension [1]. Contemporary guidelines including the National Institute for Health and Care Excellence (NICE), Kidney Disease Improving Global Outcomes (KDIGO), and European Society of Hypertension (ESH) guidelines [2–4] provide detailed guidance on accurate BP measurement. The randomised controlled trials referred to in these guidelines were all based on outpatient clinic BP (sometimes referred to as office BP) measured according to a standardised method. This standardised BP method involves 3 domains: patient preparation, environment and BP measurement technique [5].

Non-standardised BP measurement can lead to over or under-estimation of BP of unpredictable magnitude and this will inevitably lead to sub-optimal treatment [6]. For this reason, international guidelines emphasise that treatment of BP should be based on standardised office BP measurement [7]. It is recognised that incorporation of all facets of standardised methodology into routine practice is challenging. The aim of this study was to determine if outpatient clinics in which BP is routinely measured adhere to the domains of standardised BP measurement. We also aimed to determine how ambulatory blood pressure measurement (ABPM) and home blood pressure monitoring (HBPM) are utilised in these clinics. In addition we determined if body mass index (BMI) is assessed at these clinics because of the notable association of overweight and obesity with hypertension [8].

## Subjects and methods

This is a descriptive cross-sectional study. Data collection was done through dissemination of an online questionnaire designed by Webropol (Helsinki, Finland). The questionnaire is a self-administered semi-structured questionnaire. A complete copy of the questionnaire and auto-generated results are available in the Data Supplement section. All regular secondary care outpatient clinics in the disciplines of cardiology, diabetes and endocrinology (D&E), general medicine (GM) and nephrology at 15 separate hospital or clinic sites were identified through the electronic patient management system. These were filtered manually to identify the outpatient clinics where it was anticipated that BP measurement is routine. The clinician responsible for a clinic was emailed and invited to complete the online questionnaire for that particular clinic. Therefore, each clinician might be invited to complete more than one questionnaire. The clinics were within the National Health Service (NHS) Greater Glasgow and Clyde region, covering a mixed urban and rural population of approximately 1.2 million including seven hospitals with 15 separate outpatient clinic sites.

Data analysis was done utilising Microsoft Excel Version 2310 (Microsoft, Redmont, WA, USA). Each question was extracted using automatic extraction by Webropol into an Excel file. Questions from the same domain were merged as appropriate. Ethical approval was granted by the Research Ethics Committee of the University of Glasgow’s College of Medical, Veterinary, and Life Sciences on 11th July 2023 (reference number 200220359).

## Results

### General characteristics of study participants

**Fig. 1:**
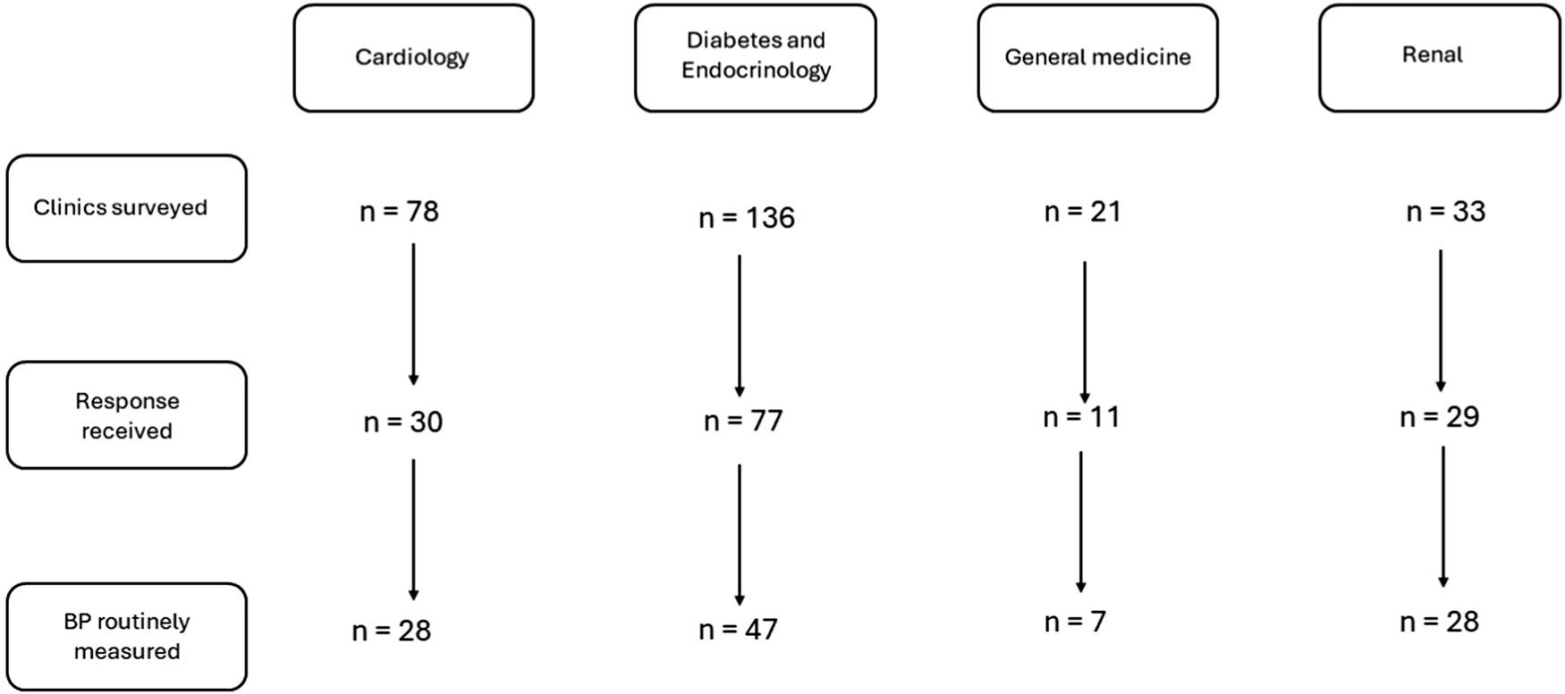
Flowchart of survey responses.

Completed questionnaires were received from the supervising clinician of 110 of the 268 surveyed clinics (41%). Table 1 shows general characteristics from these clinics. Participating clinics were divided into four groups i.e., cardiology, D&E, GM, and renal. The D&E group made up 42.7% of the total participants with cardiology, GM and renal having 25.4%, 6.4%, and 25.4% respondents, respectively. Figure 1 shows detailed participating clinics in this study.

**Table 1:** Characteristics of Clinics that Routinely Measure Blood Pressure.

How frequently is blood pressure measured at this clinic?		*n* (%)
At every visit		58 (79.4)
Only at first visit/new patient		4 (5.5)
Others	Annual	4 (5.5)
	Ad hoc	4 (5.5)
	Separate screening session	2 (2.7)
Not applicable	Only single visit for each patient	1 (1.4)
**Total**		73 (100)
**Clinic frequency**		
Daily		1 (1.4)
Twice per week		1 (1.4)
Weekly		60 (82.2)
Fortnightly		5 (6.8)
Thrice per month		1 (1.4)
Monthly		5 (6.8)
**Total**		73 (100)
**Approximate number of patients attending each clinic**		
<10		23 (31.5)
10–20		36 (49.3)
>20		14 (19.2)
**Total**		73 (100)
**Who takes BP measurement?**		
Nurses		26 (35.6)
Doctors		3 (4.1)
Health care assistants		10 (13.7)
Others	Pharmacist	10 (13.7)
	Physiologist	1 (1.4)
	Transplant coordinator	1 (1.4)
Doctor and nurses		4 (5.5)
Nurses and health care assistant		17 (23.3)
Doctor and health care assistant		1 (1.4)
**Total**		73 (100)

Nearly 70% of participating clinics routinely measure BP. Examples of clinics where BP is not routinely measured included highly specialised clinics e.g., clinics dedicated for echocardiography, osteoporosis, allergy, thyroid, virtual clinics and specific diabetes clinics where recent BP readings from primary care are routinely available.

### General characteristics of outpatient clinics where BP is routinely measured (*n* = 73)

Table 1 summarises characteristics of clinics that routinely measure BP (*n* = 73). Nearly 80% of these clinics measure BP at every visit. The vast majority run the clinics weekly (82.2%). Nearly half of the clinics have 10 to 20 patients in each visit (49.3%). Measurement of BP is predominantly done by nurses (35.6%), health care assistants (13.7%), and a combination of nurses and health care assistants (23.3%). In the clinics where the supervising clinician is a pharmacist (*n* = 10), nearly all BP measurement is taken by the pharmacists themselves.

### Standardised BP method (Patient preparation, environment, BP measurement technique)

Table 2 summarises the responses in relation to the domains of standardised BP measurement in the 73 respondents where BP is routinely measured. Only 3 (4.1%) clinics adhered to all facets of the standardised BP method. These were all renal clinics.

**Table 2:** Blood Pressure Measurement in Clinics.

Patient preparation	Advice on BP measurement preparation	*n* (%)
	Yes	
	- By mail/letter - By mail/letter AND at (preceding) clinic - By phone and at (preceding) clinic	1 (1.4) 3 (4.1) 1 (1.4)
	No	68 (93.1)
	**Total**	73 (100)
	**Application of standardised resting period**	**n (%)**
	Always	24 (32.9)
	Never	33 (45.2)
	Sometimes	16 (21.9)
	**Total**	73 (100)
**Environment**	**Dedicated quiet area without disturbance**	
	Yes	61 (83.6)
	No	12 (16.4)
	**Total**	73 (100)
**BP measurement technique**	**Cuff is placed around patient’s bare upper arm**	
	Yes	50 (68.5)
	No	8 (11.0)
	Unknown	15 (20.5)
	**Total**	73 (100)
	**The use of appropriate cuff size**	
	Yes	46 (63.0)
	No	9 (12.3)
	Unknown	18 (24.7)
	**Total**	73 (100)
	**Measure BP on both arms at the first visit**	
	Yes	7 (9.6)
	No	58 (79.4)
	Unknown	8 (11.0)
	**Total**	73 (100)

Advice such as avoiding exercise, caffeine, and or tobacco consumption for 30 minutes prior to BP measurement, wearing clothing that allows easy access to bare upper arms are not provided by majority of the clinics (93.1%). Clinics that provide such advice send this information by mail or letter or give this advice at preceding clinics.

The majority of respondents (*n* = 61 [83.6%]) reported having a dedicated quiet area without disturbance to measure BP. The advised standardised resting period is five minutes in a quiet area. Just over 30% of clinics apply this standardised resting period. With regard to BP measurement technique, 68.5% of participating clinics always place the cuff around the patient’s bare upper arm and 63.0% vary the cuff size according to the upper arm circumference. A large majority of clinics (79.4%) do not measure BP on both arms at the first visit.

### Reporting techniques and equipment

Nearly one-third of the participating clinics take more than one sitting BP measurement from the same arm (27.4%). If not reporting the average, various approaches are used to report BP measurement results, i.e., report all, the highest, exclude the first one if significantly higher, discard the first, and choose the lowest, or the second measurement.

Results from BP measurements are documented mostly in the electronic patient record (42.5%), both letter (non-auto-generated) and electronic patient record (26.0%), or non-auto-generated letter (24.6%). Complete data are presented in Table S1 of Data Supplement.

47.9% of respondents were able to report the types and brands of blood pressure equipment used in the clinics. The majority of clinics reported the use of an automated BP machine (71.4%) followed by a combined approach using automated machine and manual measurement with sphygmomanometer (20%). The remaining clinics use manual BP measurement (8.6%). Omron and Omicron are the two most common brands used by participating clinics. Other reported brands are Wellch Allyn, Mindray, Dinamap, and Criticare.

### Standing and lying BP

Table 3 summarises the standing and lying BP measurements. Nearly all participating clinics (98.6%) use sitting as the default position when measuring BP. Standing BP is always measured in two cardiology clinics (2.7%). Lying BP is always measured in two clinics (2.7%); one D&E and one GM clinic.

**Table 3:** Standing and Lying Blood Pressure Measurement.

Standing BP routine	Indications (if applicable)	*n*		%
Always		2		2.7
Never		28		38.4
Sometimes		43		58.9
	Postural hypotension/orthostatic hypotension		35 (81.4%)	
	Ad hoc based on doctor request		6 (13.9%)	
	Others (endocrine reasons such as steroid replacement -- hypoadrenalism, phaeochromocytoma)		2 (4.6%)	
Total		73		100
**Lying BP routine**				
Always		2		2.7
Never		43		58.9
Sometimes		28		38.4
	Postural hypotension/orthostatic hypotension		21	
	Ad hoc based on doctor request		3	
	Others (endocrine reasons, semi-recumbent)		2	
	Not specified		2	
**Total**		73		100

Most clinics conduct standing and lying BP measurement in specific circumstances (58.9% and 38.4%, respectively). Suspicion of postural hypotension such as postural dizziness, collapse or syncope were the commonest reported indications for standing or lying BP measurement.

### HBPM and ABPM

Nearly 70% of participating clinics reported that HBPM and/or ABPM are used in their clinics to complement clinic BP measurement. 21 (28.8%) use both HBPM and ABPM, 22 (30.1%) use only HBPM and 8 use only ABPM (11%).

### BMI

More than half (54.8%) of the participating clinics routinely assess BMI in every visit. Several clinics indicated that BMI assessment is done only in certain circumstances such as new patients, imaging purposes (body surface area), or if there is an impression of substantial weight change. Complete data is presented in Table S2 of Data Supplement.

## Discussion

This is the most comprehensive survey of outpatient clinic BP measurement practice in routine secondary care settings reported to date. Although guidelines describe the standardised BP method, there is no quality assurance system in place to ensure that recorded results have been performed by a standardised method unlike, for example, laboratory data. Our data confirmed previous reports that routine outpatient clinic BP measurement rarely adheres to the standardised method that was used in clinical trials of blood pressure treatment and is recommended in international guidelines, with only 4.1% of the surveyed clinics where BP is routinely measured adhering to all facets of the standardised method. This has important implications as all treatment decisions in the remaining clinics are potentially flawed by misinterpretation of the BP measurements taken in a non-standardised way. Previous studies indicate that the difference between non-standardised and standardised BP measurement for an individual patient is unpredictable and can be >40 mmHg [9, 10]. It is notable that 70% of the respondents use HBPM and ABPM to complement outpatient clinic BP measurement but treatment decisions based on these BP measurement techniques have less certainty than treatment decisions based on standardised BP measures [2–4].

The lack of patient preparation by most of the clinics means that there is a risk of over-estimation of BP due to patients having consumed caffeine, exercised or have a sensation of a full bladder, while smoking tobacco or other inhalants might artificially lower BP. Failure by the majority of clinics to measure BP in a dedicated quiet area after at least 5 minutes of rest will lead to overestimation of BP. Failure to measure in both arms at the first visit to determine which arm has the higher BP and should be used for future clinic BP measurement may lead to falsely low BP readings in the future. Approximately 10% of patients with chronic conditions such as kidney disease have inter-arm BP difference of >10 mmHg [11]. Failure to ensure that BP is always taken on the bare arm with the correct cuff size for that arm leads to both over-estimation and under-estimation of BP [12].

Previous studies that have explored barriers to standardised method being used for routine BP measurement at clinics reported time pressure (busy clinics), pressure to maintain clinician workflow and limited resources including clinic layouts as important factors [13, 14]. These factors were likely prominent explanations for the lack of adherence in the clinics we surveyed although this was not the focus of our study. Our survey, in a health service for a population of 1.2 million with hundreds of patients attending clinics every week where BP measurement and related treatment decisions are routine, indicates that the lack of adherence to standardised BP method means that patients are having inadequate treatment of BP and, at this scale, this has major resource implications related to consequences of both under and over-treatment of BP. It is likely that investment to ensure standardised BP measurement was applied at all these clinics would be trivial compared to these consequences and therefore would be highly cost-effective [15, 16]. In terms of HBPM and ABPM, the vast majority of the participating clinics conduct these measures when the office reading is high. This shall not erase the fact that when office BP measurements are not done correctly, over- or underestimation might still occur.

Our study had strengths and limitations. This is a large survey covering several secondary care specialities across all secondary care sites in a defined health service area. In addition to that, this study also highlights the fact that renal clinics tend to adhere to all facets of BP measurement. This is particularly important considering the relationship between kidney diseases and blood pressure. Out of three renal clinics that adhered to all facets of BP measurement, two of them also conduct HBPM. This showed further adherence to the guidelines by renal clinics. One of the limitations of this study is the 41% survey return rate and the study would benefit from a higher response rate. Nevertheless, it is unlikely that our survey missed many clinics where BP is routinely measured as we targeted specialties where BP management is a major focus of care. It seems likely that the clinics that did not respond were either equally or less likely to adhere to the standardised method. The fact that the survey was performed in a single health service area means that the result might not be generalizable. This seems unlikely as there are reports in the literature of the challenges of implementing the standardised method and discussion at national and international meetings indicates this is a widespread issue [17, 18]. We described adherence to the standardised method but did not explore the barriers. Future studies should explore these barriers including clinician education, patient education real estate infrastructure, staffing resource and equipment and lead to studies of how to implement the necessary changes. This might need novel solutions e.g., if resources are limited it would be better to measure BP by the standardised method every alternate visit for regular attendees than by the non-standardised method every visit. A similar study should also be done in primary care where a large proportion of blood pressure management is conducted in the UK.

Our comprehensive survey demonstrated very low adherence to all facets of the standardised BP method in secondary care clinics where BP measurement is routine. This has important implications for the quality of treatment decisions. Clinicians should pay close attention to how BP is being measured before making treatment decisions. We believe that greater priority should be given to the quality of BP measurement in outpatient settings in a similar way to quality controls relating to other routine clinical measurements such as laboratory measures.

## Summary

### What is known about the topic

- Blood pressure (BP) measurement is a common procedure conducted in various disciplines and widely available on clinical reports.
- The challenges in application of standardised method for BP measurement in routine clinical practice are often overlooked.

### What this study adds

- A description of BP measurement techniques across NHS Greater Glasgow and Clyde (population 1.2 million).
- In a wide range of secondary care out-patient clinic settings, BP measurement rarely adheres to the recommended standards.

## Supplementary information

Data supplement

Questionnaire

## Data Availability

Data are available from the corresponding author on reasonable request.
