# Supplementary material for: Blood pressure measurement technique in clinical practice in the NHS Greater Glasgow and Clyde
Source: J Hum Hypertens. 2024 Dec 5;39(3):205–9. doi: 10.1038/s41371-024-00984-5 (PMC11893439; doi:10.1038/s41371-024-00984-5)
Supplement: Supplementary file 1 — Data supplement [file 41371_2024_984_MOESM1_ESM.docx]

**Data Supplement**

| **More than one measurement*** |  | **n (%)** |  |  | **n (%)** |
| --- | --- | --- | --- | --- | --- |
| **Yes** | 2 | 9 (12.3) | **Is the average calculated and recorded?** | Yes | 9 (45) |
|  | 3 | 9 (12.3) |  | No | 10 (50) |
|  | >3 | 1 (1.4) |  | No data | 1 (5) |
|  | No data | 1 (1.4) |  | Total | 20 (100) |
|  | Total | 20 (27.4) |  |  |  |
| **No** | | 53 (72.6) |  | | |
| Total | | 73 (11) |  |  |  |
| **Documentation Methods** | | | **n (%)** | | |
| Letter (non-auto-generated) | | | 18 (24.6) | | |
| Electronic patients’ records | | | 31 (42.5) | | |
| Other – patients sticker sheet (+ dictated letter); SCI Diabetes | | | 2 (2.7) | | |
| Letter (non-auto-generated) AND Electronic patients’ records | | | 19 (26.0) | | |
| Letter (non-auto-generated) AND Other – letter to GP | | | 2 (2.7) | | |
| Electronic patients’ records AND Other (case-notes) | | | 1 (1.4) | | |
| Total | | | 73 (99.9) | | |

Table S1. BP measurement Variations and Reporting | *in one sitting from the same arm.

| **Variables** | | **n (%)** |
| --- | --- | --- |
| **Body weight** | Routinely in most patients | 58 (79.4) |
|  | Never | 8 (11) |
|  | Other – please specify:   - If BMI is looking very high (2) - New patient (1) - Imaging purpose (index to BSA) (1) - No details (3) | 7 (9.6) |
| **Total** | | 73 (100) |
| **Body height** | Routinely in most patients | 40 (54.8) |
|  | Never | 14 (19.2) |
|  | Other – please specify:   - New patient (11) - Imaging purpose (index to BSA) (2) - No details (4) - Young adult (2) | 19 (26.0) |
| **Total** | | 73 (100) |
| **BMI** | BMI routinely measured | 40 (54.8) |
|  | Weight only | 9 (12.3) |
|  | BMI in new patient/1st visit | 10 (13.7) |
|  | Never | 8 (11.0) |
|  | Weight and Height for imaging (BSA) | 2 (2.7) |
|  | Sometimes BMI | 2 (2.7) |
|  | If BMI looking very high | 2 (2.7) |
| **Total** | | 73 (99.9) |

Table S2. Body Weight, Height, and BMI Measurements in Participating Clinics
