# Supplementary material for: Blood pressure measurement technique in clinical practice in the NHS Greater Glasgow and Clyde
Source: J Hum Hypertens. 2024 Dec 5;39(3):205–9. doi: 10.1038/s41371-024-00984-5 (PMC11893439; doi:10.1038/s41371-024-00984-5)

Assessing the Clinical Practice in NHS GGC Outpatient Clinics for Blood Pressure Measurement

☐ Mandatory questions are marked with a star (*)

Thank you for agreeing to take part in our Blood Pressure Measurement Audit

This survey aims to assess how blood pressure measurements are done across different secondary care clinics in NHS GGC.
We are aware that there is a wide range of clinics with different requirements for blood pressure readings. Our survey will collect this information so that we can understand if blood pressure measurement can be improved. It will also support interdisciplinary integration to assess cardiovascular risk factors.

The first question of this survey serves as a consent form that must be read carefully. Should you agree to participate, please complete this consent page by ticking the boxes and typing your initial.

We appreciate how precious your time is. That's why we have included just a few essential questions, that should take you no more than 10-15 minutes in total to complete.
Be assured that the data we collect will only be used for the purposes of this research.

Thanks very much for your participation. Greatly appreciated and let's get started!

1. DIGITAL CONSENT FORM *

☐ I confirm that I have read and understood the Participant Information Sheet version 1.0 dated 07/07/2023.

☐ I confirm that I have read and understood the Privacy Notice version 1.0 dated 07/07/2023.
I have had the opportunity to think about the information and ask questions, and understand the answers I have been given.

☐ I understand that my participation is voluntary and that I am free to withdraw at any time, without giving any reason, without my legal rights being affected.

- [ ] I confirm that I agree to the way my data will be collected and processed and that data will be stored for up to 2 years in University archiving facilities in accordance with relevant Data Protection policies and regulations.
- [ ] I understand that all data and information I provide will be kept confidential and will be seen only by the study researchers and regulators whose job it is to check the work of researchers.
- [ ] I agree that my name, contact details and data described in the information sheet will be kept for the purposes of this research project.
- [ ] I understand that if I withdraw from the study, my data collected up to that point will be retained and used for the remainder of the study.
- [ ] I agree to take part in the study.
- [ ] Please type your initial to indicate participation ______

2. Please enter your clinic details *

Clinic name and code (mentioned in the email) ______________________

Responder's name ______________________

Responder's contact (email/phone) ______________________

3. Does this clinic routinely measure blood pressure? *

- ○ Yes
- ○ No

4. Please give a brief description about this clinic and its routine activities: *

____

____

____

____

____

5. What is the frequency of this clinic? *

- ○ Weekly
- ○ Every two months
- ○ Monthly
- ○ Every three months
- ○ Every six months
- ○ Other, please describe

6. What is the average number of patients attending each instance of this clinic *

- ○ <10
- ○ 10-20
- ○ >20

7. For an individual patient, how frequently is blood pressure measure at this clinic? *

- ○ At every visit
- ○ Only at first visit/new patient
- ○ Other (please describe) ____________

8. Who measures the blood pressure at this clinic? (please tick as appropriate) *

- ☐ Nurse(s)
- ☐ Doctor(s)
- ☐ Health care assistant(s)
- ☐ Others (please describe) ____________

9. Are patients advised how they should prepare (themselves) for blood pressure measurement before they come to this clinic?

***Examples of ideal preparation for blood pressure measurement: advice to avoid exercise, food, tobacco for 30 mins, advice to wear clothing that allows easy access to bare upper arms. ***

- ○ No
- ○ Yes (prior to visit)

10. How do patients receive information about the blood pressure measurement preparations? (tick as appropriate) *

- ☐ By phone
- ☐ By email
- ☐ By main/letter
- ☐ Others (please describe) ____________

11. Is a standardised resting period* before blood pressure measurement applied when blood pressure is being measured at this clinic?

***e.g., 5 minutes in a quiet area ***

- Always
- Never
- Sometimes; please specify under which circumstance(s): ____________

12. Is the default position for measurement of blood pressure at this clinic done with the patient sitting? *

- Always
- Never
- Sometimes; please specify under which circumstance(s): ____________

13. Is standing blood pressure routinely measured at this clinic?

- Always
- Never
- Sometimes; please specify under which circumstance(s) ____________

14. Is lying blood pressure routinely measured at this clinic? *

- Always
- Never
- Sometimes; please specify under which circumstance(s): ____________

15. Which device is used to measure blood pressure at this clinic?
Please provide as much information as possible (brand, type, manufacturer, etc.) *

- Please answer here: ____________
- I do not know

16. Is blood pressure measured in a quiet area without disturbance when blood pressure is measured at this clinic? *

- Yes
- No
- I do not know

17. Is blood pressure measurement always done with the cuff around the patient's bare upper arm at this clinic? *

- ◯ Yes
- ◯ No
- ◯ I do not know

18. Is the cuff size selected according to the patient's upper arm circumference at this clinic? *

- ◯ Yes
- ◯ No
- ◯ I do not know

19. Is blood pressure measured in both arms at the patient's first visit to this clinic to establish which arm blood pressure should be measured from in the future? *

- ◯ Yes
- ◯ No
- ◯ I do not know

20. Is more than one sitting blood pressure measurement taken from the same arm when blood pressure is measured at this clinic? *

- ◯ No
- ◯ Yes

21. How many times blood pressure is measured for each patient (each arm) in one visit?

- ◯ 2
- ◯ 3
- ◯ >3

22. Is the average of these blood pressure measures calculated and recorded?

- ◯ Yes
- ◯ No

23. How are the results from blood pressure measurements processed?

Examples: report all, discard the first and do an average of second and third, only the third

24. Which documentation methods are used to record blood pressure measurements results? (tick as appropriate) *

- ☐ Letter (non-auto-generated)
- ☐ Electronic patients records
- ☐ Other (please describe)

25. Are other methods of blood pressure measurement used at this clinic? (tick as appropriate) *

- ☐ Home Blood Pressure Monitoring (HBPM)
- ☐ Ambulatory Blood Pressure Monitoring (ABPM)
- ☐ Other (please specify)
- ☐ None

26. Is patient body weight measured at this clinic? *

- ○ Routinely in most patients
- ○ Never
- ○ Other (please explain)

27. Is patient height measured at this clinic so that body mass index can be calculated? *

- ○ Routinely in most patients
- ○ Never
- ○ Other (please explain)

Thank you for your participation - please click the Submit button to finish.

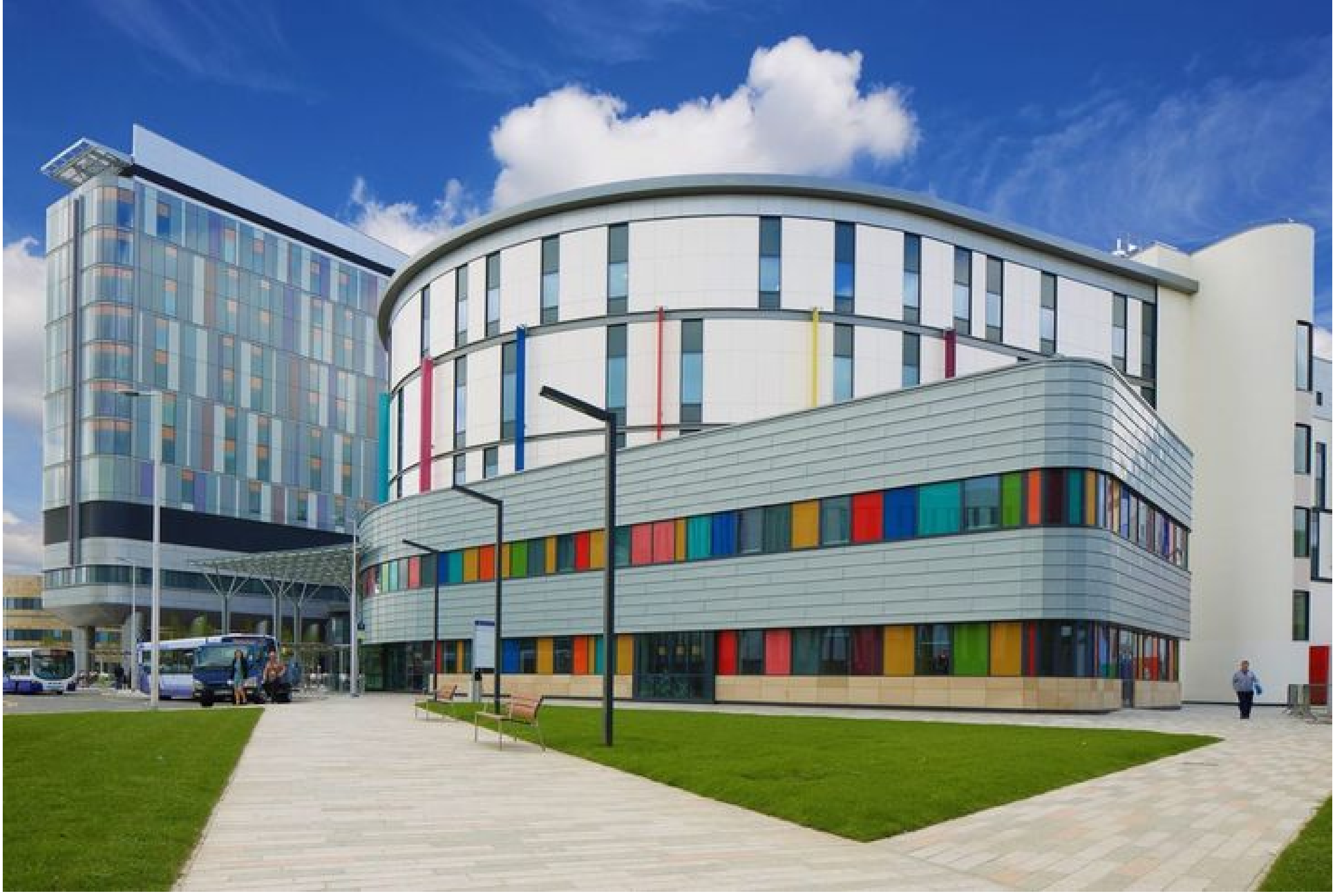

Supplement: Supplementary file 2 — Questionnaire [file 41371_2024_984_MOESM2_ESM.pdf]
